# Supplementary material for: Profiling Plant circRNAs Provides Insights into the Expression of Plant Genes Involved in Viral Infection
Source: Life (Basel). 2025 Jul 20;15(7):1143. doi: 10.3390/life15071143 (PMC12298914; doi:10.3390/life15071143)
Supplement: Supplementary file 1 [file life-15-01143-s001.zip › life-3671640-supplementary.pdf]

Supplementary Material

Morphological symptoms resulting from virus infection or expression of a single viral gene in *Arabidopsis* plants.

Figure S1.

**Sample**	**Symptoms description**	**Plant 1**	**Plant 2**
RYMV (transgenic)	No Visible symptoms	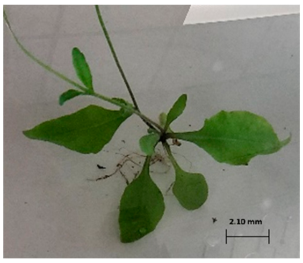	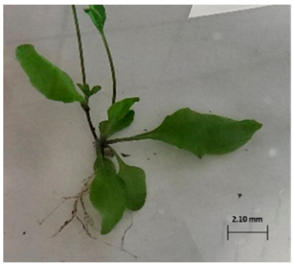
CP-TRoV (transgenic)	Some Black spots	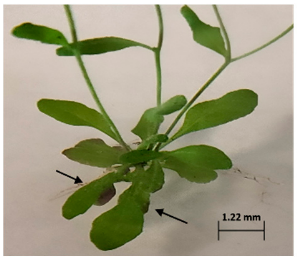	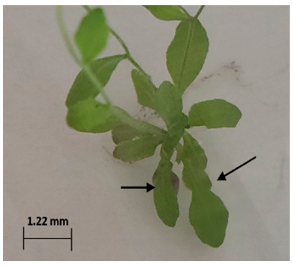
circ-LTSV (transgenic)	No visible symptoms	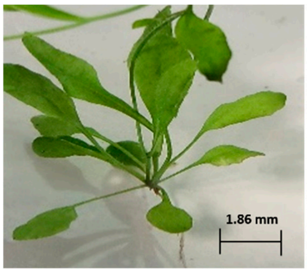	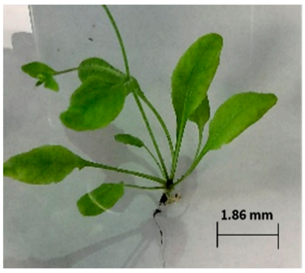
CP-TRoV + sc-LTSV	No visible symptoms	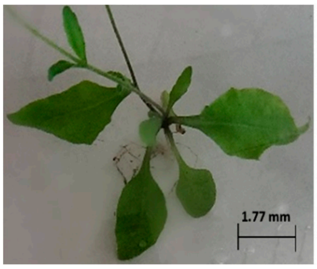	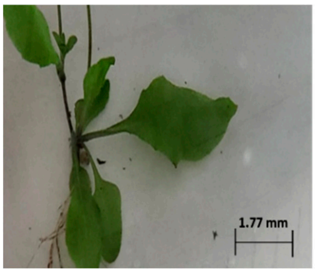

10 dpi (TRoV)	White spots on the lower and upper leaves	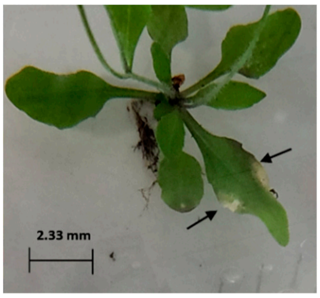	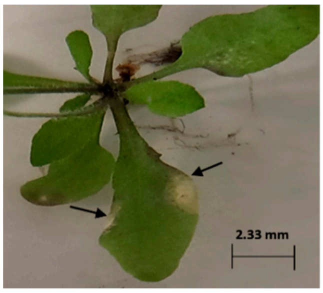
15 dpi (TRoV)	Purple spots and dry leaves	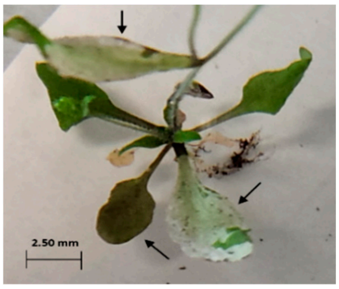	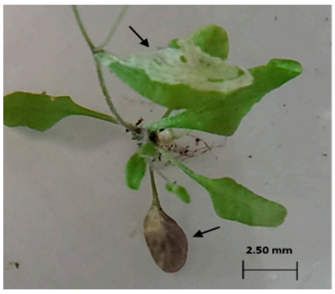
Healthy	No visible symptoms	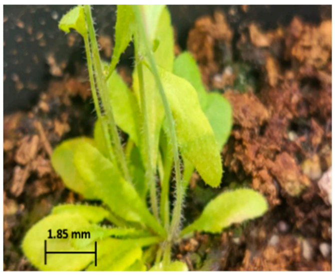	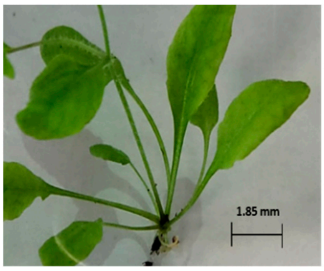

2. **Construction of the pBI121 CP-TRoV clone.**

Previous work from our laboratory generated a construct in which the CP-TRoV and scLTSV were co-cloned into the binary vector pBI121 (unpublished results, Ahmad and AbouHaidar, 2016). To examine whether any of the endogenous circRNAs can play a role in host-virus recognition and interaction, the above construct was used to generate a clone containing only the CP-TRoV gene after removing the scLTSV from this construct.

This CP-TRoV-scLTSV construct contained CP-TRoV cloned between the Xba I and Sac I restriction sites of pBI121 and the scLTSV cloned at the downstream Sac I site (Fig. S2).
In order to generate transgenic A. thaliana expressing only the CP-TRoV, the above construct was digested with Sac I wherein in a 20 reaction, 1 µg of the above plasmid DNA was digested with 10 U of Sac I restriction endonuclease using 1x Cut Smart buffer for 4 hours at 37 C° to remove the scLTSV, leaving the CP-TRoV in the clone after ligation. The SacI-digested

plasmid DNA was electrophoresed in a 1% TBE-agarose gel to separate it from the released scLTSV genome (322 nucleotides) and the ~14 kb plasmid DNA containing the CP-TRoV alone was extracted from the gel using the Qiagen DNA extraction kit. Following purification, the Sac I-digested DNA was ligated back using T4 DNA ligase as follows: 2.5 µl of 10X T4 DNA Ligase buffer, 5 µl linearized, purified plasmid (100 ng), 1 µl T4 DNA ligase (400 units), and 16.5 µl nuclease-free water in a 25 µl reaction. Ligation was performed overnight at room temperature.

This construct contained the natural start and stop codons of the CP-TRoV along with the 3' untranslated (3' UTR) region. The integrity of the above clone was substantiated by nucleotide sequencing and by PCR using primers specific for both the CP-TRoV (an 800 nt PCR product) and the scLTSV (no PCR product) (see the supplemental Fig. S4) which showed the successful removal of scLTSV in the final clone while retaining only the CP-TRoV.

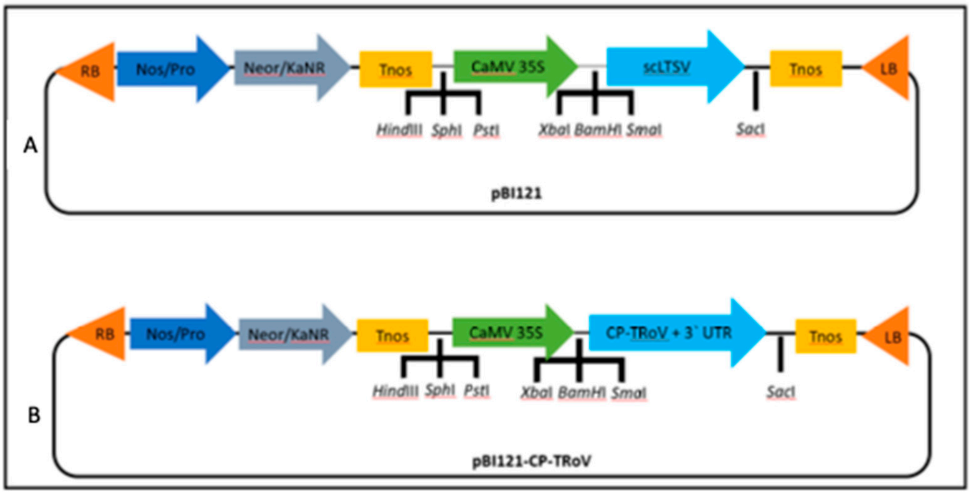

Figure S2. Schematic representation of CP-TRoV construct. The TRoV capsid protein is cloned between Xba I and Sac I restriction sites of pBI121. All elements are downstream of the cauliflower mosaic virus (CaMV) 35S promotor are destined for transcription, ending at the nopaline synthesis terminator (Tnos). TRoV possesses independent translation start and stop codons. The top part of the images shows the CP-TRoV followed by the scLTSV cloned into pBI121 vector, and the lower part shows the CP-TRoV cloned into the pBI121 plasmid.

3. **Construction of pCambia 1300 (scLTSV-TRoV).**

A head-to-tail sequence of the 322-nt LTSV satellite along with CP-TRoV had been previously constructed in the pCambia 1300 plasmid in our laboratory, and its identity was confirmed by sequencing.

The CP-TRoV was removed by Xba I digestion, and the remaining plasmid DNA containing the scLTSV was ligated back (Fig. S3). The Xba I digested plasmid DNA was electrophoresed in a 1% TBE-agarose gel and the ~9 kb plasmid DNA containing the circ-LTSV was extracted from the gel using the Qiagen DNA extraction kit. Following the purification, the XbaI

digested DNA was ligated back using T4 DNA ligase as follows: 2.5 µl of 10X T4 DNA Ligase buffer, 5 µl linearized, purified plasmid (100 ng), 1 µl T4 DNA ligase (400 units), and 16.5 µl nuclease-free water in a 25 µl reaction. Ligation was performed overnight at room temperature.

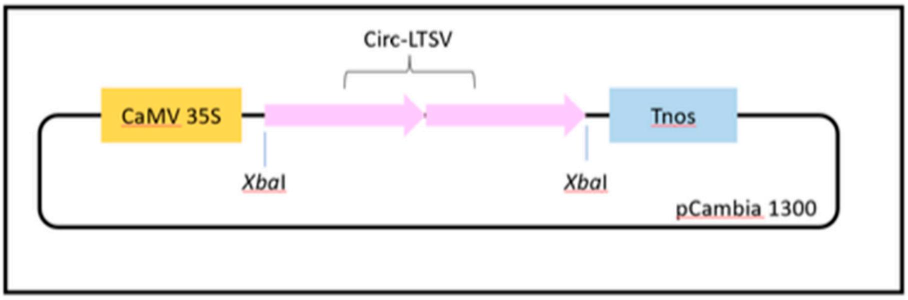

Figure S3. Schematic representation of scLTSV constructs. The circ-LTSV insert is cloned at the XbaI site, producing both forward (F) and reverse (R) orientations. All elements downstream of the cauliflower mosaic virus (CaMV) 35S promoter are destined for transcription, ending at the nopaline synthase terminator (Tnos).

4. Construction of pCambia 1300 (RYMV) clone.

To generate the transgenic A. thaliana expressing the RYMV genome, RYMV infectious clone that was generated in our laboratory in pUC19 (Venkataraman and AbouHaidar, 2019) was cloned into pCambia 1300 downstream of the 35S promoter (Fig. S4). This design was chosen to enhance the expression of the RYMV genome in the non-host A. thaliana, facilitating its utilization for investigating circRNA profiles induced by RYMV genomic RNA in *A. thaliana*.

This RYMV infectious genome construct was cloned into pCambia 1300 Xba I site using both forward and reverse primers derived from the 1-25 nucleotides of either end of the RYMV genomic sequence to amplify the RYMV infectious construct followed by cloning of this 4.5 kb PCR product into the Xba I site of pCambia 1300. The pCambia 1300 vector was digested with Xba I as follows: in 20 µl reaction, 1 µg of the above plasmid DNA was digested with 10 U of Xba I restriction endonuclease using Cut Smart buffer for 4 hours at 37 C°.

The Xba I digested plasmid DNA was electrophoresed in a 1% TBE-agarose gel and the ~ 14 kb plasmid DNA containing the RYMV was extracted from the gel using the Qiagen DNA

extraction kit. Following the purification, the Xba I digested DNA was ligated back using T4 DNA ligase as follows: 2.5 µl of 10X T4 DNA Ligase buffer, 5 µl linearized, purified plasmid (100 ng), 1 µl T4 DNA ligase (400 units), and 16.5 µl nuclease-free water in a 25 µl reaction. Ligation was performed overnight at room temperature.

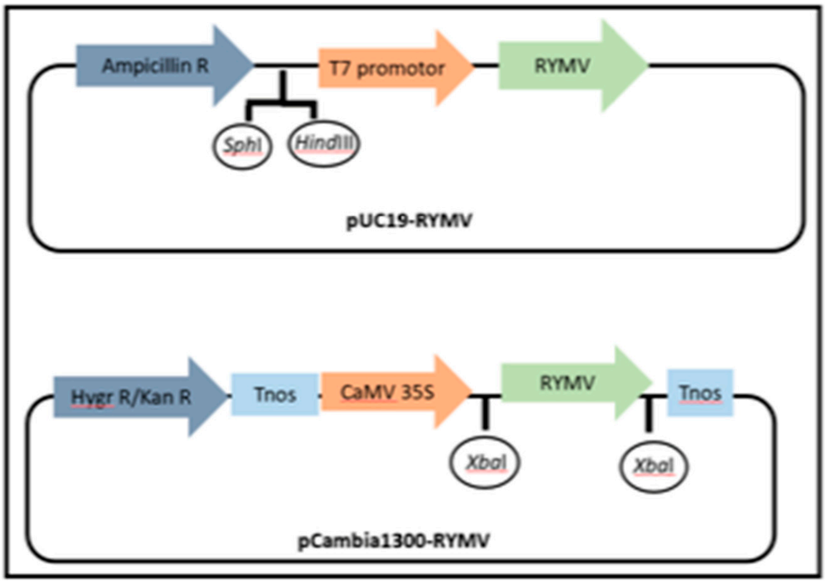

Figure S4. Schematic representation of RYMV construct. The forward and reverse primers for the RYMV PCR product contain Xba I site. All elements are downstream of the cauliflower mosaic virus (CaMV) 35S promotor are destined for transcription, ending at the nopaline synthesis terminator (Tnos). The top part of the figure shows the RYMV cloned into pUC19 vector, and the lower part shows the RYMV cloned into the pCambia1300 plasmid

5. **Confirmation of the successful cloning of the CP-TRoV gene into binary vector pBI121.**

We performed the successful cloning of the CP-TRoV gene in the binary vector pBI121and confirmed the identity of the clone by performing PCR amplification using primers specific to the CP-TRoV, which showed the expected 800 bp band (Fig. S5) while at the same time showing the absence of any product using primers specific to the scLTSV, thus authenticating the CP-TRoV construct.

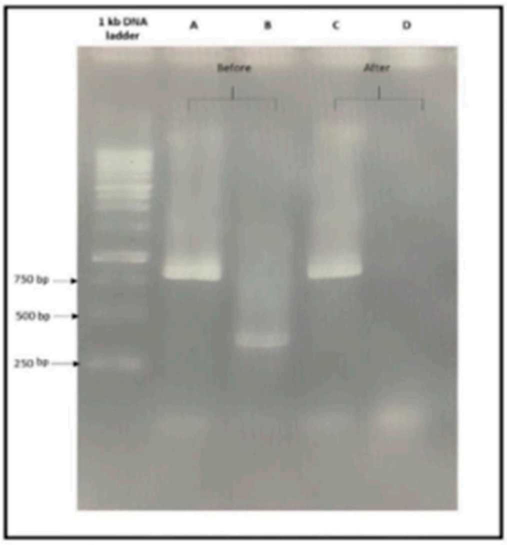

Figure S5. Confirmation of cloning of the CP gene of TRoV into the pBI121 vector by PCR amplification. Lanes A and B show the previously generated construct in our lab; lane A shows the 800 bp CP-TRoV band and lane B shows the 332 bp scLTSV band from the construct before removing the scLTSV. Lanes C and D show PCR of the construct after removing the scLTSV; lane C shows the amplification of the CP-TRoV alone and not the scLTSV (lane D), thus proving the cloning of the CP-TRoV alone. Lane 1 represents 1 kb DNA ladder.

6. **Confirmation of the successful cloning of the scLTSV into binary vector pCambia1300**.

To examine whether the (exogenous) circRNA of the scLTSV virusoid, either by itself or in combination with the TRoV helper virus, has any impact on the plant endogenous circRNA profile or other plant regulatory processes, we cloned the circ-LTSV form of scLTSV in pCambia 1300 (Fig. S6).

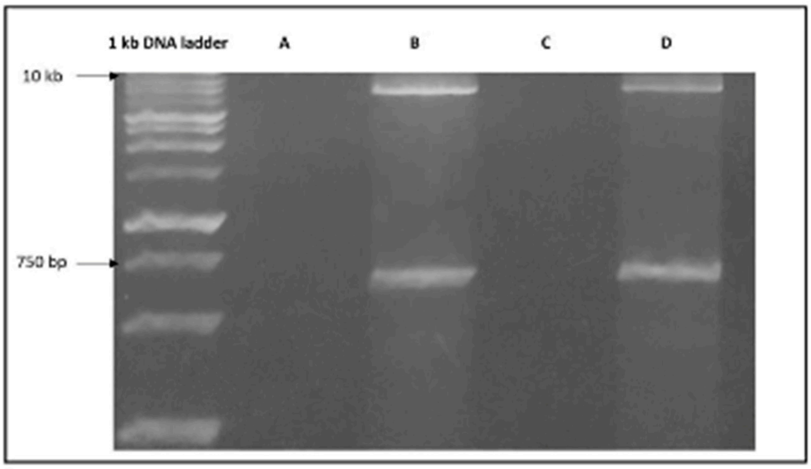

Figure S6. Confirmation of cloning of the circ-LTSV into the pCambia 1300 vector through PCR amplification. The first lane displays a 1 kb DNA ladder, while Lanes B and D show bands corresponding to circ-LTSV RNA (~600 bp). The upper bands in both lanes represent the binary vector pCambia 1300 with a size of 8.9 kb. Lanes A and C are left intentionally empty.

7. **Confirmation of the successful cloning of the RYMV genome into pCambia1300.**

The RYMV cannot be propagated in A. thaliana as it is a non-host plant for RYMV. To identify the gamut of endogenous circRNAs that are under- / over-expressed in viral host versus non-host systems, the infectious clone of RYMV (generated in this laboratory) was overexpressed under the control of the transcriptionally strong 35S-promoter in transgenic *A. thaliana* (Fig. S7).

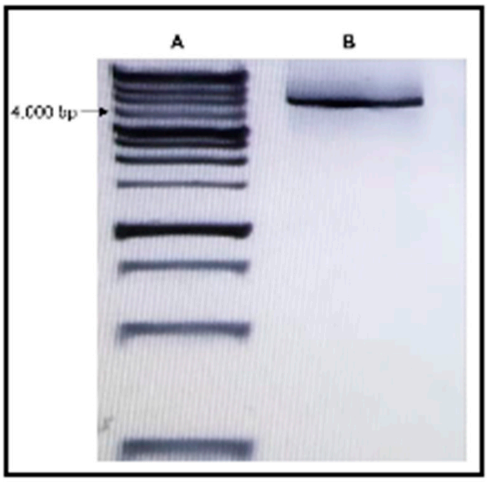

Figure S7. Confirmation of cloning of the RYMV genome into the pCambia 1300 vector by PCR amplification. Lane A: 1 kb DNA ladder. Lane B: 4.500 bp RYMV sequence (complete genome).

Transgenic plants were generated as described in the material and method section, following which the plants were processed to verify the expression of the respective transgenes in these plants.

8. Confirmation of transgenic *A. thaliana* plants.

Generation of A. thaliana plants transgenic for CP-TRoV, RYMV and sc-LTSV (Fig. S8).

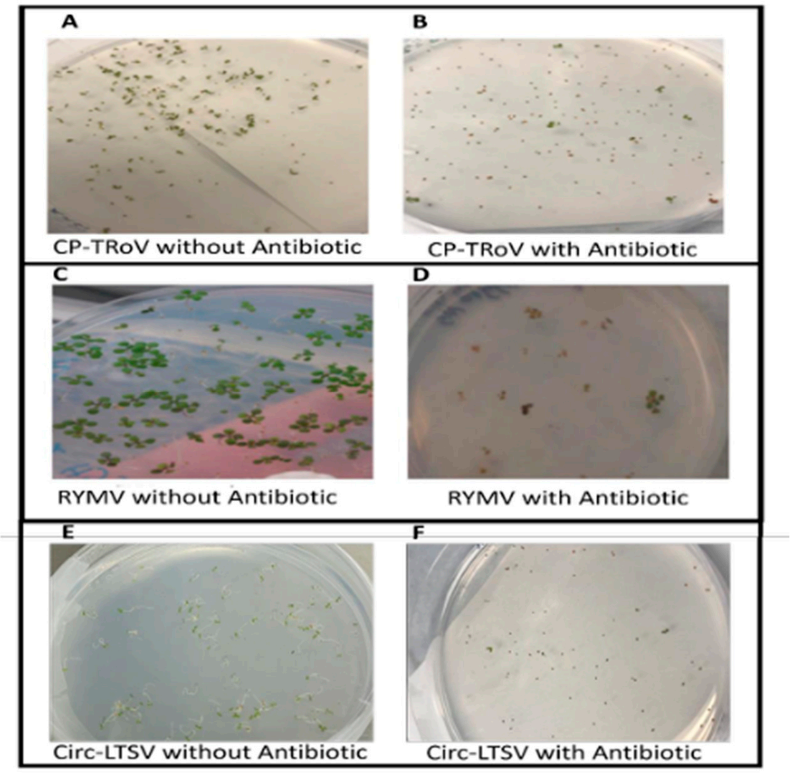

Figure S8. Screening of CP-TRoV, genomic RYMV and genomic circ-LTSV transgenic seeds. Panel A, transgenic CP-TRoV A. thaliana were screened ½ MS medium without antibiotics as a control. Panel B trans- genic CP-TRoV *A. thaliana* were screened on 20 µg/mL Hygromycin in ½ x MS medium. Panel C, transgenic genomic RYMV A. thaliana plants were screened ½ MS medium without antibiotics as a control. Panel D transgenic genomic RYMV A. thaliana was screened on 20 µg/mL Hygromycin in ½ x MS medium. Panel E transgenic genomic circ-LTSV A. thaliana were screened ½ MS medium without antibiotics as a control. 20 µg/mL Hygromycin in ½ x Panel F transgenic genomic circ-LTSV A. thaliana plants were screened on MS medium.

9. Electrophoretic analysis of the integrity of total RNA preparations used as templates for generating circular RNAs

Next, we checked the integrity of our total RNA preparations as shown in Fig. S9. Figure S9, panel A group 1 (total nucleic acid samples) shows the top bands representing genomic DNA in all samples (4 lanes); group 2 shows purified total RNA after removal of the genomic DNA following DNase treatment. Group 3 shows controls treated with water instead of DNase under the same conditions as those for Group 2. (Keren et al., 2011), and there was no noticeable degradation in all the RNA samples, thus authenticating the integrity of our total RNA samples (Fig. S9, panel A).

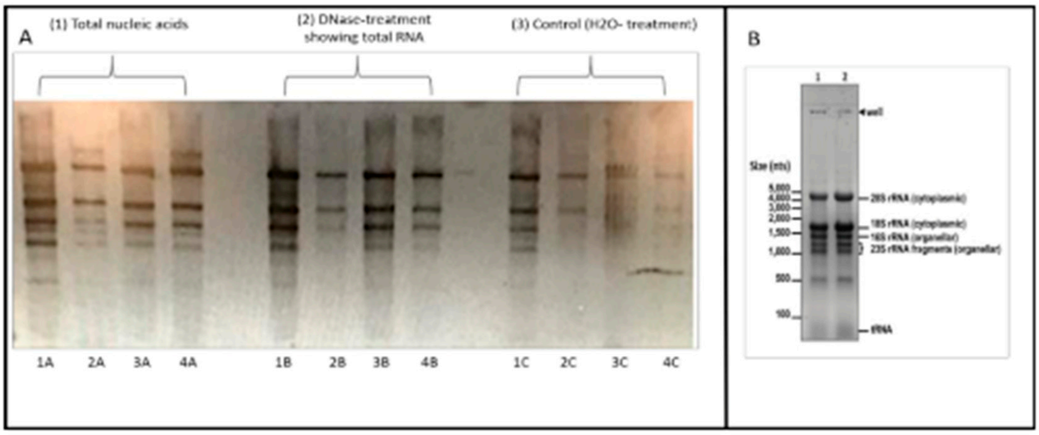

Figure S9. Demonstration of the integrity of total RNA isolated from Arabidopsis thaliana using RNA-grade agarose gel. Panel A. group 1: Total nucleic acids (without DNase treatment) healthy A. thaliana (1A), healthy turnip (2A), infected A. thaliana with TRoV (3A), and total nucleic acids from transgenic A. thaliana (4A) respectively; all samples in this group have genomic DNA contamination (top bands). Group 2: Total nucleic acids samples treated with DNase healthy A. thaliana (B1), healthy Turnip (2B), TRoV infected A. thaliana plant (3B) and total nucleic acids from transgenic A. thaliana (4B) respectively; the genomic DNA bands have been digested in this group (top bands). Group 3: control samples (H2O treated (instead of DNase) (mock-inoculated) with the same time of incubation and temperature as the samples in group B) healthy A. thaliana (1C), healthy turnip (C2), TRoV infected A. thaliana plants (3C), and total nucleic acids from transgenic A. thaliana (4C), respectively. Panel B. Integrity and size distribution of total RNA isolated from Arabidopsis leaves, analyzed by formaldehyde agarose electrophoresis gel. Adapted from Keren et al., 2011.

10. Bradford assays to ascertain equal amounts of proteins loaded for Western analyses of CP-TRoV and RYMV transgenic samples

For Western analysis, we loaded equal amounts of proteins in all the respective lanes following protein estimation through the Bradford assay. Fig. S10 shows the Bradford assay results for proteins loaded in the CP-TRoV Western analysis and Fig. S11 shows the Bradford assay results for the proteins used in the RYMV Western analysis.

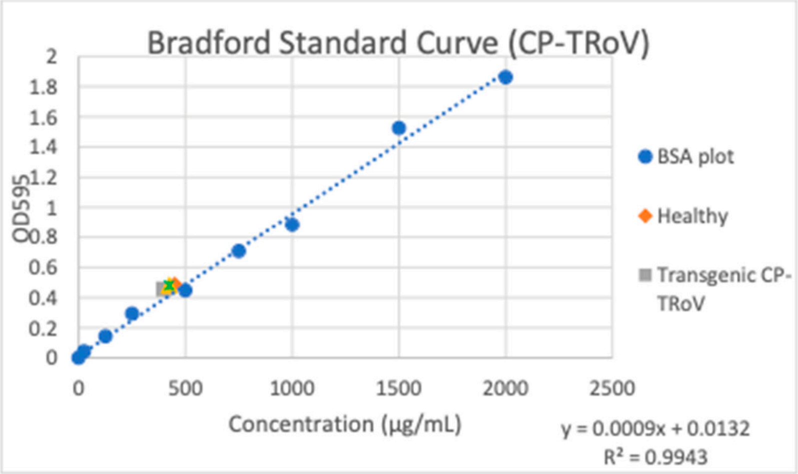

Figure S10. Bradford assay showing concentrations of the respective TRoV-infected and transgenic CP-TRoV protein samples (µg/mL). Each sample was incubated at room temperature for 5 minutes before being subjected to spectrophotometric measurement. Using BSA standard, Bradford Assay standard protein curve is shown in the above graph. The deduced concentrations of protein extracts of healthy *A. thaliana*, empty vector transgenic A. thaliana, A. thaliana transgenic for CP-TRoV and A. thaliana infected with TRoV are also shown.

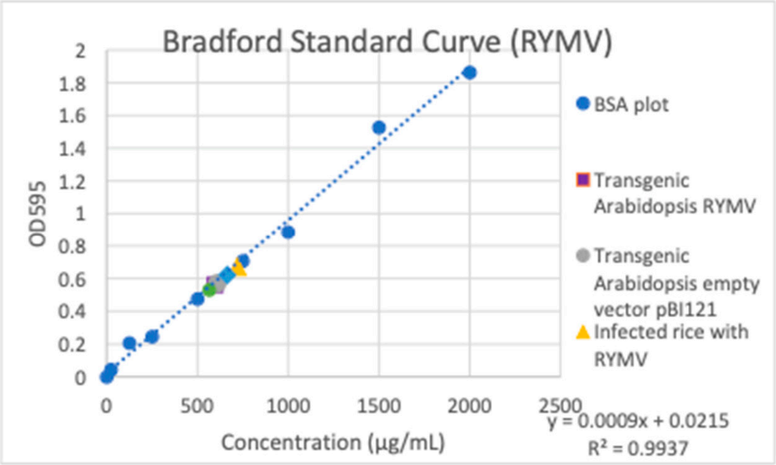

Figure S11. Bradford assay showing concentrations of protein samples (µg/mL) in RYMV transgenic plants. Each sample was incubated at room temperature for 5 minutes before being subjected to spectrophotometric measurement. Using BSA standard, Bradford Assay standard protein curve is shown in the above graph. The deduced concentrations of protein extracts of *A. thaliana* transgenic for RYMV, empty vector transgenic *A. thaliana*, and rice infected with RYMV are also shown.

11. Primer validation for RT-qPCR analyses

As part of primer validation for RT-qPCR, standard curves were generated using a 10-fold serial dilution of pooled cDNA. Primer efficiencies were calculated using the slope of each curve, and only those with efficiencies between 90% and 110% and R^2 values above 0.98 were accepted for analysis. Representative standard curves for selected primers are shown in Figure S12, demonstrating high efficiency (94–102%) and excellent linearity ($R^2 > 0.99$).

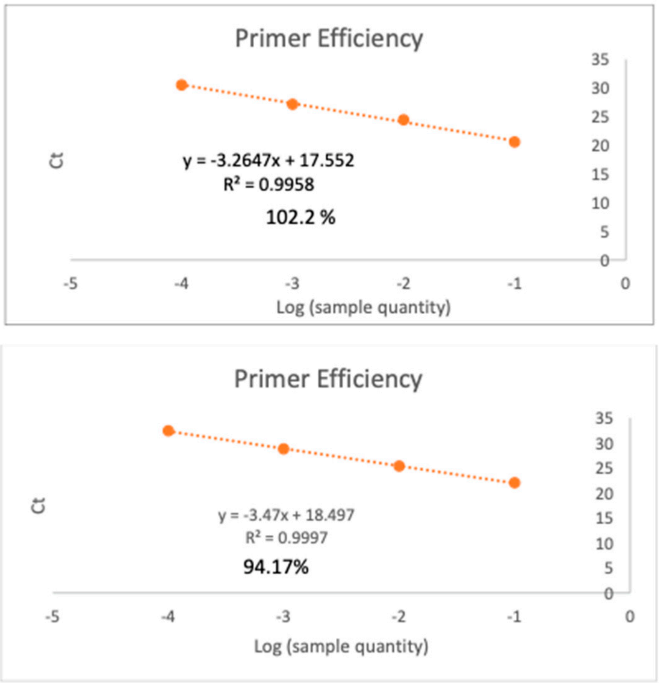

Figure S12. Some examples of Standard curves: Cq is calculated from ct values are plotted (Y-axis) against the log of the copy number (x-axis) of the template to establish a standard curve with an efficiency of 102.2% from the slope -3.2647 with R2 value 0.9958 and efficiency of 94.17 % from the slope -3.347 with R2 value 0.9997. Each standard curve represents an individual primer.

12. RT-PCR validation of selected dysregulated circRNAs

Figure S13 presents RT-PCR validation of selected dysregulated circRNAs using divergent and convergent primers, confirming the circular nature of the transcripts through the presence or absence of specific amplification products on 2% agarose gels.

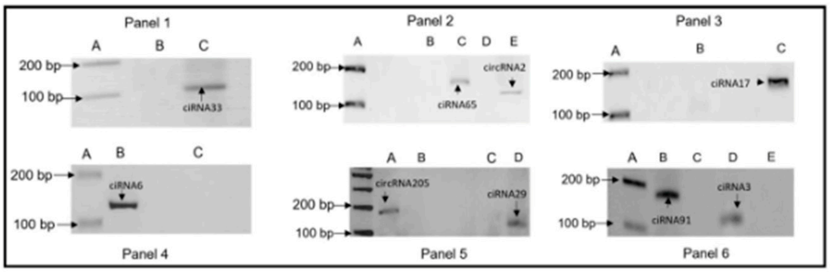

Figure S13. RT-PCR verification of the selected dysregulated circRNAs in A. thaliana samples using 2% agarose gel. From left to right. Panel 1: Lane C: RT-PCR product (115 bases) from ciRNA33 using divergent primers. Panel 2: Lanes C and E, respectively: RT-PCR products from ciRNA65 (167 bases) and circRNA2 (142 bases) using divergent primers. No PCR products are showing in lanes B and D with the convergent primers. Panel 3: Lane C: RT-PCR product primers. Panel 4: Lane B: RT-PCR product (135 bases) from ciRNA6 using divergent primers, while no product shows in lane C using convergent primers. Panel 5: Lanes A and D: RT-PCR products from circRNA205 (195 bases) and circRNA29 (125 bases) using divergent primers. No PCR products are showing in lanes B and C with the convergent primers. Panel 6: Lanes B and D, respectively: RT-PCR products from ciRNA91 (196 bases) and circRNA3 (134 bases) using divergent primers. No PCR products are showing in lanes C and E with the convergent primers. 100 bases DNA ladder in all lanes A.

13. Supplemental Tables

To evaluate the potential effects of viral infection and transgene expression on plant growth, the height of six individual Arabidopsis thaliana plants per treatment group was measured. **Table S1** summarizes the plant height data along with the calculated standard deviations, illustrating variability within each sample group.

Table S1. The plant height in all *A. thaliana* samples utilized in this study. Observed between the six different plants within each sample.

Sample name	Plant 1 length (mm)	Plant 2 length (mm)	Plant 3 length (mm)	Plant 4 length (mm)	Plant 5 length (mm)	Plant 6 length (mm)	Standard Deviation (mm) ±
RYMV	23.8	20.1	18.9	22	18	20.6	2.10
CP-TRoV	15.4	14.8	13.2	13.6	16.2	13.4	1.22
CP-TRoV +sc-LTSV	18.7	16.5	20	19.5	17.3	15.5	1.77
Circ-LTSV	18	20.7	16.3	17.8	21.2	19	1.86
Healthy	22.5	24.4	25.1	20.8	23	20.5	1.85
10 dpi (TRoV)	17.5	16.9	20.5	22	16.9	17.8	2.13
15 dpi (TRoV)	20.7	18.8	14.8	21	17.2	20.9	2.50

Total RNA concentration and purity were assessed before and after DNase treatment for all samples using spectrophotometry. **Table S2** presents the RNA yield and quality (OD260/OD280), confirming that all RNA samples used in downstream applications were of acceptable purity and concentration.

Table S2. Spectrophotometer readings of total RNA (before and after DNase treatment) samples.

Sample	Total RNA before DNase treat-ment		Total RNA after DNase treat-ment.	
Healthy (negative control)	Concentra-tion (ng/µl)	OD260/ OD280	Concentra-tion (ng/µl)	OD260/ OD280
	49.4	2.0	41.2	1.8
Infected with TRoV (positive control)	105.3	2.0	73.3	1.9
Transgenic CP-TRoV	36.4	1.9	24.5	1.8
Healthy Rice (negative control)	58.2	2.0	52.2	1.9
Mocked-inoculated	45.2	2.0	41.2	1.8
Infected rice with RYMV	210.2	2.0	179.2	2.0
Transgenic genomic RYMV	51.4	1.9	43.2	1.8
Healthy turnip	40.2	2.0	32.5	1.8
Infected Turnip with LTSV	89.2	1.9	65.2	1.8
Transgenic scLTSV	28.2	1.9	22.6	1.8

Table S3 shows the relative expression levels of selected circRNAs, calculated using the 2^–ΔΔCt method after normalization to the elongation factor reference gene, confirming differential expression patterns observed in the RNA-seq analysis.

Table S3. Relative quantification of circRNAs expression of some of the selected dysregulated circRNAs using real-time RT-PCR.

Sample	Ct (mean)		ΔCt		ΔΔCt	2- ΔΔCt
	Target gene	EF Housekeeping gene	Treated sample	Untreated sample		Target difference relative control
circRNA247	27.1	24.3	2.8	5.5	2.7	0.15
circRNA248	30.8	24.3	6.5	6.7	-0.16	1.12
circRNA221	28.3	24.3	3.9	4.5	0.57	0.67
circRNA296	34.1	23.05	11.1	11.9	-0.7	1.7
CP-TRoV (circRNA205)	32.8	23.18	9.6	10.7	-1.09	2.1
RYMV (circRNA205)	26.6	22.3	4.2	6.5	-2.3	4.9
circRNA 205 (circ-LTSV)	33.8	22.1	11.6	7.4	4.2	0.5
circRNA236	34.1	23.05	11.1	11.9	-0.79	1.7
ciRNA11	25.3	23.03	2.3	9.6	7.2	0.01
circRNA103	31.22	24.7	6.4	6.7	-2.0	4.09
circRNA95	31.1	24.5	6.5	5.5	-1.0	0.1
ciRNA9	17.5	22.7	-5.3	9.6	14.8	3.3×10^{-5}
circRNA25	34.2	23.04	11.2	9.5	1.6	0.3
circRNA3	31.8	22.8	9.0	6.2	2.8	0.1
ciRNA17	33.0	22.1	10.8	6.2	4.6	0.04

Total RNAs were extracted and normalized to the expression of endogenous reference elongation factor gene. Threshold cycle (Ct): the number of cycles at which the fluorescence exceeds the threshold.; mean values of duplicate assays carried out with different samples. The values for the targeted genes are the average for 3 replicates. ΔCt: Difference between values of reference and target (target is normalized to the reference). ΔΔCt: ΔCt of each sample is further normalized to the control. 2-ΔΔCt: Fold change relative to control.

References

Keren, Ido & Abudraham, Sivan & Shaya, Felix & Ostersetzer-Biran, Oren. (2011). An optimized method for the analysis of plant mitochondria RNAs by Northern-blotting.
